# Alcohol-Related Birth Defects—The Past, Present, and Future

**Published:** 2001

**Authors:** Kenneth R. Warren, Laurie L. Foudin

**Affiliations:** Kenneth R. Warren, Ph.D., is director of the Office of Scientific Affairs, National Institute on Alcohol Abuse and Alcoholism (NIAAA) and Laurie L. Foudin, Ph.D., is program director for fetal alcohol research in the Division of Basic Research, NIAAA, Bethesda, Maryland

**Keywords:** Fetal alcohol effects, fetal alcohol syndrome, birth defects, alcohol-related neurodevelopmental disorder, diagnostic criteria, risk factors, AODR (alcohol- or other drug-related) biological markers, targeted prevention, warning label

## Abstract

In 1994 Alcohol Health & Research World (now titled Alcohol Research & Health) last devoted a full issue to the topic of fetal alcohol syndrome (FAS) and other alcohol-related birth defects (ARBD). This introductory article provides readers with information on how the field has advanced since then. In addition to tracing the development of the terminology used in the field, it describes the difficulties involved in determining the true prevalence of FAS and ARBD; the mechanisms that may play a role in alcohol-derived fetal injuries; approaches to preventing drinking during pregnancy; and strategies for assisting people who have been born with FAS and ARBD.

In 1994 *Alcohol Health & Research World* (now titled *Alcohol Research & Health*) last devoted a full issue to the topic of fetal alcohol syndrome (FAS) and other alcohol-related birth defects (ARBD) (see Volume 18, Number 1, 1994). Selected by the National Association of Government Communicators as first prize in the technical publications category, that issue of the journal provided an excellent overview of the existing knowledge on alcohol-derived prenatal injury and still remains a valuable resource for readers. Since the publication of that issue, however, research in the ARBD field has advanced significantly. This current issue of *Alcohol Research & Health* presents a selection of these new, important findings.

In the interval between the two publications on ARBD, the United States Congress directed the National Institute on Alcohol Abuse and Alcoholism (NIAAA) to prepare a comprehensive report on FAS. In response, NIAAA commissioned the Institute of Medicine (IOM) of the National Academy of Sciences to conduct a study. The resulting seminal report, *Fetal Alcohol Syndrome: Diagnosis, Epidemiology, Prevention, and Treatment* (Stratton et al. 1996*a*), critically reviewed the major scientific issues in fetal alcohol research. Among its many recommendations, the IOM Committee proposed a revision of the terminology used regarding manifestations of alcohol-derived prenatal injury, particularly because the terminology is very much interwoven with the clinical and scientific issues related to the diagnosis of FAS and other alcohol-related prenatal effects. The new terminology was designed to better meet the needs of various constituencies, including policymakers, scientists, clinicians, and other health care providers who deal with these issues.

The term “fetal alcohol syndrome” was introduced in 1973 by Jones and Smith (1973), whose original diagnostic criteria have changed very little even after being reconsidered by other groups, such as the Fetal Alcohol Study Group of the Research Society on Alcoholism (Rosett 1980; Sokol and Clarren 1989). However, after the FAS diagnostic criteria were introduced, it became clear that there were people who likely had been adversely affected by prenatal alcohol exposure but who did not completely fulfill the criteria for a diagnosis of FAS. One term that had been introduced to include such cases was “fetal alcohol effects” (FAE) (Clarren and Smith 1978). But, unlike the term “FAS,” not all clinicians and researchers used the term “FAE” uniformly. Consequently, the IOM addressed this confusion by introducing more refined definitions, which have helped to provide consistency in the terminology used to describe the problems caused by prenatal alcohol exposure. For this reason, it is worthwhile to review the diagnostic criteria in the IOM report in some detail.

The IOM developed five diagnostic categories (see textbox). The first two pertain to FAS itself. The other categories address various aspects of the spectrum of alcohol-related disorders. Researchers previously had disagreed whether an FAS diagnosis could be made without evidence of maternal alcohol use. Some investigators had argued that the phenotype (i.e., the visible characteristics) of FAS appeared to be sufficiently unique to permit the diagnosis to be made even in the absence of information on maternal drinking; other investigators, however, felt uncomfortable about making an FAS diagnosis without a confirmation of maternal drinking. The issue of drinking history as one of the diagnostic criteria is important, because maternal drinking history is frequently unknown. Because no validated objective biological marker currently exists to confirm maternal drinking during pregnancy or alcohol exposure of the fetus (although researchers are trying to find one; see the article in this issue by Bearer, pp. 210–218), researchers must rely on maternal self-reports as well as reports from the mother’s collateral acquaintances. However, many affected children are in foster care or adopted, and accurate drinking information for the birth mother, even from her collaterals, is not readily available for these children.

**IOM-Recommended Diagnostic Criteria for FAS and Alcohol-Related Effects**

**Category 1. FAS With Confirmed Maternal Alcohol Exposure**

1. Confirmed maternal alcohol exposure*
2. Characteristic pattern of facial anomalies, including short palpebral fissures, and abnormalities of the premaxillary zone (e.g., flat upper lip, flattened philtrum, flat midface)
3. Growth retardation, such as low birth weight, lack of weight gain over time, disproportional low weight to height
4. Neurodevelopmental abnormalities of the CNS, such as small head size at birth; structural brain abnormalities with age-appropriate neurological hard or soft signs (e.g., impaired fine motor skills, neurosensory hearing loss, poor tandem gait, poor eye-hand coordination)

**Category 2. FAS Without Confirmed Maternal Alcohol Exposur**e

Characteristics 2–4 as in Category 1

**Category 3. Partial FAS With Confirmed Maternal Alcohol Exposure**

1. Confirmed maternal alcohol exposure*
2. Some components of the FAS facial pattern
  either 3, 4, or 5 below:
3. Growth retardation as in Category 1
4. CNS neurodevelopmental abnormalities as in Category 1
5. Complex pattern of behavioral or cognitive abnormalities inconsistent with developmental level and unexplained by genetic background or environmental conditions (e.g., learning difficulties; deficits in school performance; poor impulse control; problems in social perception; language deficits; poor capacity for abstraction; specific deficits in mathematical skills; and problems in memory, attention, or judgment)

**Category 4. Alcohol-Related Birth Defects (ARBD)**

1. Confirmed maternal alcohol exposure*
2. One or more congenital defects, including malformations and dysplasias of the heart, bone, kidney, vision, or hearing systems

**Category 5. Alcohol-Related Neurodevelopmental Disorder (ARND)**

1. Confirmed maternal alcohol exposure*
2. CNS neurodevelopmental abnormalities as in Category 1
  and/or
3. Complex pattern of behavioral or cognitive deficits as in Category 3

*Maternal alcohol exposure is defined as a pattern of excessive alcohol intake characterized by substantial, regular intake or by heavy episodic (i.e., binge) drinking. Evidence of this pattern may include signs of alcohol dependence.CNS = central nervous system; FAS = fetal alcohol syndrome; IOM = Institute of Medicine.SOURCE: Stratton et al. 1996*a*.

The IOM addressed this problem by creating two FAS categories that differed from each other only on whether maternal alcohol exposure could be confirmed. At-risk maternal drinking during pregnancy was defined as “a pattern of excessive intake characterized by substantial, regular intake or heavy episodic drinking” (Stratton et al. 1996*a*, p. 77). Several indicators of this type of risk drinking were listed, including evidence of withdrawal episodes and of social or legal problems attributable to drinking. These are also indicators of alcohol dependence. If all other diagnostic requirements are present for FAS except confirmation of maternal alcohol exposure, cases could be assigned to category 2. However, if confirmation existed that risk drinking did *not* occur, a diagnosis of FAS would not be made, even if the affected person appeared to have all the signs of FAS.

The other elements of the FAS diagnosis in the IOM definitions do not deviate significantly from the original descriptions provided by Jones and Smith (1973) and Clarren and Smith (1978). These elements include evidence of growth retardation (e.g., low birth weight, lack of weight gain over time, or a low weight-to-height ratio); evidence of neurodevelopmental abnormalities (e.g., a small-sized brain [i.e., microcephaly] or other structural brain abnormalities); and a characteristic pattern of mild facial anomalies, including small eye openings (i.e., short palpebral fissures), a thin upper lip, or flattened ridges between the base of the nose and the upper lip (i.e., a flattened philtrum). (See figure.)

The other three IOM diagnostic categories describe conditions that do not meet the FAS criteria. All require a confirmation of substantial maternal alcohol use because the phenotypes for these diagnoses are not considered unique enough to be ascribed to prenatal alcohol exposure without evidence of maternal drinking.

Category 3 includes partial FAS with confirmed maternal alcohol exposure—in other words, some, but not all, of the facial characteristics required for an FAS diagnosis must be present as well as confirmed evidence of maternal alcohol exposure. In addition, at least one of the three following indicators also must be present: growth deficits normally characteristic of FAS, neurodevelopmental abnormalities, or behavioral and cognitive problems consistent with those observed in FAS. The latter indicator includes a complex pattern of deficits in learning, school performance, impulse control, and the cognitive functions involved in guiding behavior. Category 4 encompasses ARBD and was proposed for people with heart, bone, kidney, vision, or hearing defects who had been prenatally exposed to alcohol. Such organ abnormalities are not uncommon in FAS, although they are not observed as consistently as other FAS features. When the behavioral and cognitive problems of FAS and partial FAS are present, but the facial features are normal, the affected individual is assigned to category 5, alcohol-related neurodevelopmental disorder (ARND). Because ARND and ARBD can co-occur, a person may receive a dual diagnosis.

The terminology introduced by the IOM provides flexibility for clinical applications and more precision for research applications. However, as new knowledge about the nature of the deficits accrues, further refinements in the defining categories will likely occur.

Among other terminology that has been introduced since the IOM study, Riley and colleagues use the term “prenatal exposure to alcohol (i.e., PEA)” in their research (e.g., Riley et al. 1995) to describe children who have been exposed to alcohol prenatally without the specific requirement for the presence of any particular deficit. Streissguth and O’Malley (2000) proposed the term **“**fetal alcohol spectrum disorders**”** (FASD) for inclusion in the *Diagnostic and Statistical Manual of Mental Disorders* (DSM-IV). FASD describes the full range, from mild to severe, of disturbances of physical, behavioral, emotional, and/or social functioning attributable to in-utero alcohol damage. These terms may prove useful in some circumstances, provided that conflicting definitions for FAS, ARBD, and ARND are not introduced.

## Determining the Prevalence and Risk Factors

One of the biggest challenges in determining the true prevalence of FAS and the associated disorders is how to recognize the syndrome, which depends in part on the age and physical features of the person being diagnosed (Larkby and Day 1997). Several distinct screening tools have been proposed to assist in making an FAS diagnosis (Astley and Clarren 1995, 1996, 2000; Burd and Martsolf 1989).

Investigators have used three different approaches in attempting to measure the prevalence of FAS: passive surveillance systems, clinic-based approaches, and active case ascertainment in a segment of the general population. May and Gossage discuss in their article in this issue, pages 159–167, both the advantages and disadvantages of these approaches. Based on recent findings, May and Gossage estimate that the prevalence of FAS in the United States during the 1980s and 1990s was 0.5 to 2.0 cases per 1,000 births.

Not every woman who drinks during pregnancy will give birth to a child with FAS or even ARND. Abel (1995) estimated that 4.3 percent of heavy drinkers give birth to an FAS child. Coles (1991) reported that half of the children of heavy drinking women were not abnormal. Therefore, defining the factors that place certain women at risk of giving birth to an alcohol-affected child is a key research issue. Risk factors include maternal age (Sokol et al. 1986; Jacobson et al. 1996), socioeconomic status (Abel 1995), ethnicity (Abel and Hannigan 1995), genetic factors (Goodlett et al. 1989; Streissguth and Dehaene 1993; Rasheed et al. 1997; Su et al. 2001; Warren et al. 2001), and maternal alcohol metabolism (Chernoff 1980; Warren et al. 2001), among others. Maier and West (see their article in this issue on pages 168–174) discuss the risks associated with different drinking patterns. These studies reveal that it is not so much the total amount of alcohol that is consumed, but rather, the high number of drinks consumed at one occasion, producing a high peak blood alcohol concentration, that appears to be the greater risk factor for prenatal injury from alcohol. In fact, Jacobson and colleagues (1998) have shown that drinking expressed as average drinks per occasion is more informative than average drinks per week. They found deficits in infant performance at the level of five drinks per occasion at least once per week. However, further research is needed to evaluate the relative contributions of the various risk factors for FAS. Identification of risk factors strongly associated with alcohol-related birth outcomes could help identify high-risk pregnancies for intervention.

## Discovering the Mechanisms Involved

Clearly, no single mechanism is responsible for the array of alcohol-derived fetal injuries. However, some putative mechanisms are particularly significant in early pregnancy, such as excessive cell death in a special population of embryonic cells that give rise to facial structures and certain peripheral nerves (i.e., cranial neural crest) (Cartwright and Smith 1995; Kotch and Sulik 1992), whereas other mechanisms appear to be more significant later in pregnancy (e.g., loss of specific brain cell numbers in the cerebellum [West 1993]). On pages 175–184, Goodlett and Horn review the current theories regarding the ways in which many of these mechanisms affect the fetus through prenatal alcohol exposure.

## Preventing Prenatal Alcohol Use

The conundrum confronting efforts to prevent FAS, ARND, and ARBD is obvious to everyone who has attempted to address this issue. As the IOM noted in its report, the problem may appear simple on the surface: “Women who drink excessively while pregnant are at high risk for giving birth to children with birth defects. Therefore, to prevent these defects, women should stop drinking alcohol during all phases of pregnancy” (Stratton et al. 1996*b*, p. 1). However, many women who drink continue to do so while they are pregnant. In fact, reports indicate a disturbing trend in recent years toward increased drinking during pregnancy, especially binge drinking (Ebrahim et al. 1998, 1999). Some women may be unaware of the risks involved, whereas alcohol-dependent women may be unable to abstain. Yet, even women who are aware of FAS and ARND, and who intend to abstain from alcohol during pregnancy, may nonetheless consume alcohol in early gestation before they realize that they are pregnant.

Given the various degrees of effort needed to address the problem of drinking in pregnancy among different populations and at different levels of risk, the IOM proposed a comprehensive intervention program encompassing a spectrum of approaches. Adapting a model originally described by Gordon (1983), the IOM report (Stratton et al. 1996*a*) describes three levels of prevention. *Universal prevention* targets an entire population group and can include such components as health advisories, public service announcements, and health articles and brochures distributed through a variety of outlets. For example, NIAAA issued a health advisory in 1977 (Department of Health, Education and Welfare 1977), which was updated with the Surgeon General’s Advisory on Alcohol and Pregnancy in 1981 (Department of Health and Human Services 1981) that recommended abstinence. Another salient example of universal prevention is the Federal law (Public Law 100-690) that requires the following label warning about the dangers of drinking while pregnant on all alcoholic beverages sold in the United States: “Government warning: 1) According to the Surgeon General, women should not drink alcoholic beverages during pregnancy because of the risk of birth defects….” In the previous ARBD issue of *Alcohol Health & Research World*, Hankin (1994) described a study to measure the effect of the alcohol warning label and recently reported on the results of that research (Hankin et al. 1996*a*,*b*).

*Selective prevention* efforts target specific groups whose risks are higher than the population in general. For example, these people may reside in a community with heavy per capita alcohol use.

*Indicated prevention* targets individuals, rather than groups, known to be at high risk because of specific risk factors; for example, a person with a known drinking problem or who has previously given birth to a child with FAS. Indicated prevention efforts may encompass a range of support activities from counseling to case management. Handmaker and Wilbourne, in their article on pages 219–229, describe a low-cost, stepped-care approach that clinicians can use to intervene and prevent drinking during pregnancy.

Indicated prevention cannot be implemented until a pregnant woman who is drinking at levels that place her fetus at risk for FAS or ARND is identified. Ongoing research is helping to develop the clinical tools necessary to meet this goal. On pages 210–218, Bearer discusses efforts to identify biomarkers that could provide evidence of risk drinking as well as identify alcohol-exposed newborns. In the absence of biomarkers, structured screening questionnaires that ask a series of nonthreatening questions about alcohol use or its consequences are instrumental in clinical practice. The article by Chang, pages 204–209, reviews such tools that are now being tested in clinics.

## Assisting People Born With FAS and ARND

Children born with FAS and ARND are in critical need of interventions that can reduce the effect of their cognitive and behavioral deficits. FAS and ARND have life-long consequences, with outcomes that are often more complex than those experienced by FAS patients who are mentally retarded. In a review of the literature on adolescents and adults, Streissguth and O’Malley (2000) found evidence of mental health problems, school problems, legal difficulties, and problems with alcohol and other drugs. However, they also found that people who receive appropriate supportive services fare better with respect to secondary disabilities and life functioning than those who do not receive such services. Multiple approaches are needed, including social support, special education, behavioral and cognitive therapy, and medications.

Efforts are under way to obtain a full understanding of the specific neurocognitive functions that are impaired or spared among people with FAS and ARND. Several articles in this issue of *Alcohol Research & Health* address this type of research. Mattson and colleagues, in their article on pages 185–191, review findings from neuropsychological and brain imaging studies that describe the characteristic pattern of learning and memory deficits in FAS and ARND, whereas Kodituwakku and colleagues, on pages 192–198, look at specific deficits in cognition-based and emotion-based executive functioning in children who were prenatally exposed to alcohol. The distinctions between children with FAS and those with attention deficit hyperactivity disorder (ADHD) are described by Coles on pages 199–203. Lastly, Mennella (pages 230–234) discusses how alcohol exposure through lactation may affect aspects of child development, particularly motor development.

The new knowledge on FAS highlighted in this issue should help clinicians and researchers in developing approaches for assisting people in overcoming behavioral and cognitive deficits and thereby improve the quality of their lives. New approaches to amelioration, such as dietary supplementation (Thomas et al. 2000) and specialized therapeutic training (Klintsova et al. 2000), are being explored in animal models.

## Looking to the Future

Although great strides have been made in identifying and characterizing the physical and neurobehavioral problems of FAS and ARND, further research is needed to accomplish the following important objectives:

- Improve the clinical recognition of women’s at-risk drinking behavior before and during pregnancy
- Intervene more effectively to modify drinking behavior during pregnancy
- Develop in-utero approaches derived from basic research to prevent or minimize alcohol-induced prenatal injury
- Determine more effective ways to identify FAS and ARND across the life span, especially in infants and children
- Develop strategies to address the neurodevelopmental and learning problems of children with FAS and

ARND, including the use of appropriate behavioral and cognitive therapies, medications, and special education programs.

Multidisciplinary approaches encompassing basic laboratory animal research, human clinical research, and epidemiology will pave the way for translating scientific knowledge into practical approaches for preventing and treating FAS, ARND, and ARBD.

## Figures and Tables

**Figure:**
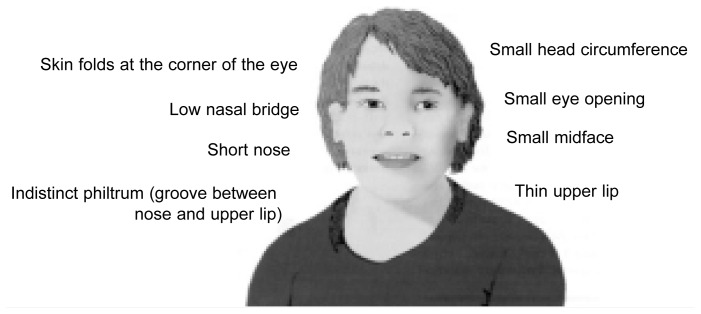
Facial features of FAS
